# Supplementary material for: Are children with disabilities more likely to be malnourished than children without disabilities? Evidence from the Multiple Indicator Cluster Surveys in 30 countries
Source: BMJ Nutr Prev Health. 2024 Jan 10;7(1):38–44. doi: 10.1136/bmjnph-2023-000779 (PMC11221280; doi:10.1136/bmjnph-2023-000779)

Supplementary Figure 1: Meta-analysis comparing prevalence of underweight in children with and without disabilities

Country	Disabled Int +	Disabled Int -	Non-disabled Int +	Non-disabled Int -	Unadjusted risk ratio [95% CI]	Adjusted risk ratio [95% CI]
Algeria	16	218	201	8654	3.59 [1.79, 7.21]	3.56 [1.80, 7.02]
Argentina	9	139	98	3745	1.19 [0.50, 2.84]	1.15 [0.48, 2.75]
Bangladesh	111	262	3328	10354	1.23 [1.03, 1.47]	1.19 [1.00, 1.41]
Central African Republic	220	564	1029	3623	1.30 [1.12, 1.50]	1.25 [1.08, 1.44]
Chad	585	939	3947	8684	1.24 [1.13, 1.36]	1.19 [1.09, 1.31]
Congo, Democratic Republic of the	316	627	3109	8689	1.28 [1.08, 1.51]	1.25 [1.06, 1.47]
Costa Rica	8	146	53	2005	2.66 [0.93, 7.57]	2.26 [0.67, 7.58]
Cuba	0	68	96	3192	0.15 [0.02, 1.24]	0.16 [0.02, 1.32]
Dominican Republic	15	186	132	4742	3.11 [1.52, 6.38]	2.67 [1.25, 5.72]
Gambia	86	331	899	4850	1.40 [1.10, 1.78]	1.32 [1.04, 1.67]
Ghana	57	494	545	4317	0.69 [0.48, 1.00]	0.65 [0.45, 0.95]
Guinea-Bissau	30	158	713	3701	0.91 [0.62, 1.32]	0.89 [0.61, 1.30]
Honduras	42	240	370	4525	2.13 [1.52, 2.99]	2.05 [1.47, 2.86]
Iraq	28	313	275	9563	3.55 [1.99, 6.31]	3.23 [1.77, 5.89]
Kiribati	19	137	56	1056	2.36 [1.40, 3.97]	2.18 [1.29, 3.68]
Lao People's Democratic Republic	84	136	1722	5236	1.49 [1.20, 1.85]	1.25 [1.01, 1.54]
Lesotho	15	154	181	1677	0.84 [0.47, 1.49]	0.80 [0.46, 1.41]
Madagascar	235	515	1898	4978	1.09 [0.95, 1.24]	1.10 [0.96, 1.26]
Malawi	91	398	1081	7563	1.38 [1.08, 1.76]	1.36 [1.06, 1.73]
Mongolia	4	85	69	3645	1.96 [0.52, 7.36]	1.83 [0.48, 7.06]
Nepal	37	41	1075	3035	2.10 [1.64, 2.69]	2.02 [1.58, 2.59]
Pakistan	1888	3691	15971	46062	1.27 [1.21, 1.33]	1.25 [1.19, 1.31]
Palestine, State of	6	82	59	3560	2.11 [0.78, 5.75]	2.03 [0.74, 5.60]
Samoa	5	110	46	1410	0.95 [0.36, 2.46]	1.03 [0.39, 2.70]
Sao Tome and Principe	3	67	58	1034	0.90 [0.26, 3.09]	0.92 [0.26, 3.21]
Sierra Leone	73	441	735	5839	1.17 [0.91, 1.51]	1.13 [0.87, 1.45]
Suriname	1	91	151	2466	0.12 [0.02, 0.88]	0.12 [0.02, 0.88]
Togo	46	180	419	2328	1.33 [0.94, 1.88]	1.34 [0.96, 1.87]
Tunisia	2	77	20	2072	3.04 [0.71, 12.95]	3.10 [0.68, 14.10]
Zimbabwe	20	129	328	3280	1.37 [0.87, 2.14]	1.28 [0.82, 2.02]
Test for heterogeneity: tau^2=0.08; chi^2=96.43, df=29, P<0.001; I^2=87%<br>Test for overall unadjusted random effect: Z=4.82, P<0.001					1.40 [1.22, 1.60]	-
Test for heterogeneity: tau^2=0.07; chi^2=86.14, df=29, P<0.001; I^2=85%<br>Test for overall adjusted random effect: Z=4.42, P<0.001					-	1.33 [1.17, 1.51]

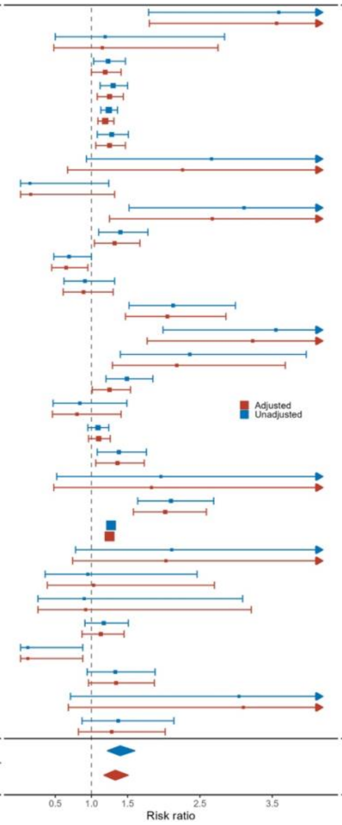

Supplementary Figure 2: Meta-analysis comparing prevalence of underweight in girls with and without disabilities

Country	Disabled Int +	Disabled Int -	Non-disabled Int +	Non-disabled Int -	Unadjusted risk ratio [95% CI]	Adjusted risk ratio [95% CI]
Algeria	5	87	89	4239	6.13 [2.17, 17.37]	6.07 [2.28, 16.13]
Argentina	5	52	52	1808	1.52 [0.45, 5.17]	1.51 [0.45, 5.07]
Bangladesh	41	108	1700	4908	1.13 [0.85, 1.50]	1.11 [0.84, 1.46]
Central African Republic	100	292	505	1842	1.24 [1.00, 1.53]	1.21 [0.98, 1.49]
Chad	289	463	1834	4384	1.27 [1.10, 1.45]	1.21 [1.06, 1.39]
Congo, Democratic Republic of the	116	294	1520	4525	1.04 [0.77, 1.41]	0.99 [0.75, 1.33]
Costa Rica	4	50	31	974	1.92 [0.56, 6.60]	1.53 [0.41, 5.68]
Cuba	0	28	41	1600	0.92 [0.11, 7.52]	0.82 [0.08, 8.45]
Dominican Republic	5	78	72	2382	3.28 [1.10, 9.76]	2.78 [0.92, 8.35]
Gambia	40	136	442	2395	1.67 [1.19, 2.36]	1.55 [1.10, 2.18]
Ghana	28	225	263	2236	0.83 [0.50, 1.38]	0.78 [0.47, 1.30]
Guinea-Bissau	11	74	345	1809	0.80 [0.42, 1.52]	0.78 [0.41, 1.49]
Honduras	14	104	168	2243	1.83 [1.02, 3.27]	1.76 [0.99, 3.13]
Iraq	20	123	181	4657	5.01 [2.60, 9.67]	4.77 [2.39, 9.55]
Kiribati	6	58	24	546	2.38 [0.96, 5.89]	2.21 [0.88, 5.59]
Lao People's Democratic Republic	45	58	862	2596	1.81 [1.37, 2.39]	1.48 [1.12, 1.94]
Lesotho	5	65	85	885	0.68 [0.24, 1.91]	0.69 [0.25, 1.93]
Madagascar	114	232	909	2516	1.25 [1.04, 1.50]	1.29 [1.07, 1.55]
Malawi	34	151	533	3855	1.18 [0.79, 1.78]	1.15 [0.76, 1.74]
Mongolia	3	37	34	1753	3.18 [0.70, 14.44]	3.38 [0.74, 15.57]
Nepal	19	18	535	1437	2.31 [1.72, 3.10]	2.13 [1.61, 2.81]
Pakistan	844	1705	7455	22658	1.28 [1.20, 1.38]	1.25 [1.16, 1.34]
Palestine, State of	5	33	23	1722	6.00 [1.89, 19.04]	7.00 [2.10, 23.28]
Samoa	3	45	21	678	1.40 [0.41, 4.78]	1.43 [0.40, 5.12]
Sao Tome and Principe	2	26	24	515	1.39 [0.33, 5.77]	1.36 [0.33, 5.65]
Sierra Leone	36	198	420	2919	0.94 [0.65, 1.35]	0.91 [0.64, 1.30]
Suriname	1	43	64	1179	0.26 [0.04, 1.95]	0.26 [0.04, 1.92]
Togo	24	69	211	1132	1.48 [0.92, 2.37]	1.43 [0.91, 2.26]
Tunisia	1	34	9	1029	3.78 [0.49, 29.04]	4.90 [0.61, 39.63]
Zimbabwe	8	55	160	1685	1.53 [0.77, 3.06]	1.41 [0.69, 2.87]
Test for heterogeneity: tau^2=0.11; chi^2=78.65, df=29, P<0.001; I^2=80%<br>Test for overall unadjusted random effect: Z=4.66, P<0.001					1.48 [1.25, 1.74]	-
Test for heterogeneity: tau^2=0.09; chi^2=73.33, df=29, P<0.001; I^2=78%<br>Test for overall adjusted random effect: Z=4.27, P<0.001					-	1.40 [1.20, 1.63]

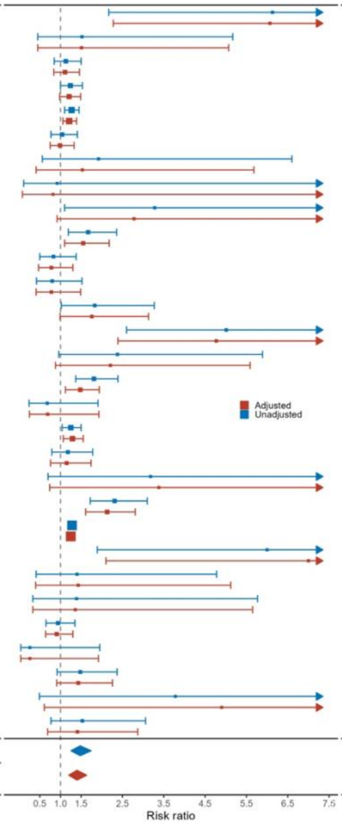

Supplementary Figure 3: Meta-analysis comparing prevalence of underweight in boys with and without disabilities

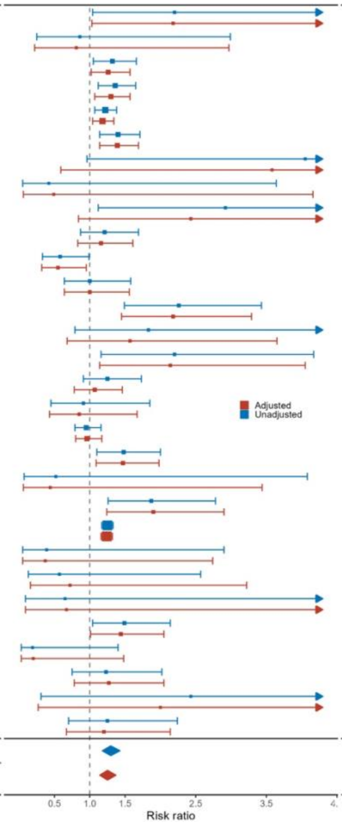

Country	Disabled Int +	Disabled Int -	Non-disabled Int +	Non-disabled Int -	Unadjusted risk ratio [95% CI]	Adjusted risk ratio [95% CI]
Algeria	11	131	112	4415	2.20 [1.04, 4.66]	2.18 [1.03, 4.59]
Argentina	4	87	46	1937	0.86 [0.25, 2.99]	0.81 [0.22, 2.97]
Bangladesh	70	154	1628	5446	1.32 [1.05, 1.66]	1.26 [1.02, 1.57]
Central African Republic	120	272	524	1781	1.36 [1.12, 1.65]	1.30 [1.07, 1.57]
Chad	296	476	2113	4300	1.22 [1.07, 1.38]	1.18 [1.04, 1.34]
Congo, Democratic Republic of the	200	333	1589	4164	1.40 [1.14, 1.71]	1.39 [1.14, 1.69]
Costa Rica	4	96	22	1031	4.05 [0.96, 17.09]	3.58 [0.59, 21.68]
Cuba	0	40	55	1592	0.42 [0.05, 3.64]	0.49 [0.06, 4.16]
Dominican Republic	10	108	60	2360	2.92 [1.12, 7.62]	2.43 [0.84, 6.99]
Gambia	46	195	457	2455	1.21 [0.87, 1.69]	1.16 [0.83, 1.61]
Ghana	29	269	282	2081	0.58 [0.33, 0.99]	0.55 [0.32, 0.95]
Guinea-Bissau	19	84	368	1892	1.00 [0.64, 1.58]	1.00 [0.64, 1.56]
Honduras	28	136	202	2282	2.26 [1.49, 3.43]	2.18 [1.45, 3.29]
Iraq	8	190	94	4906	1.83 [0.79, 4.22]	1.57 [0.68, 3.65]
Kiribati	13	79	32	510	2.20 [1.16, 4.17]	2.14 [1.14, 4.05]
Lao People's Democratic Republic	39	78	860	2640	1.25 [0.91, 1.73]	1.07 [0.78, 1.46]
Lesotho	10	89	96	792	0.91 [0.45, 1.85]	0.85 [0.43, 1.67]
Madagascar	121	283	989	2462	0.95 [0.79, 1.16]	0.96 [0.80, 1.17]
Malawi	57	247	548	3708	1.48 [1.10, 2.00]	1.47 [1.09, 1.98]
Mongolia	1	48	35	1892	0.52 [0.07, 4.08]	0.44 [0.06, 3.44]
Nepal	18	23	540	1598	1.87 [1.26, 2.78]	1.90 [1.24, 2.90]
Pakistan	1044	1986	8516	23404	1.25 [1.17, 1.33]	1.24 [1.16, 1.32]
Palestine, State of	1	49	36	1838	0.39 [0.05, 2.90]	0.37 [0.05, 2.74]
Samoa	2	65	25	732	0.57 [0.13, 2.57]	0.72 [0.16, 3.22]
Sao Tome and Principe	1	41	34	519	0.65 [0.09, 4.56]	0.67 [0.09, 4.91]
Sierra Leone	37	243	315	2920	1.49 [1.04, 2.14]	1.44 [1.01, 2.05]
Suriname	0	48	87	1287	0.19 [0.03, 1.40]	0.20 [0.03, 1.48]
Togo	22	111	208	1196	1.23 [0.75, 2.02]	1.27 [0.78, 2.05]
Tunisia	1	43	11	1043	2.43 [0.31, 18.80]	2.00 [0.27, 14.68]
Zimbabwe	12	74	168	1595	1.25 [0.70, 2.24]	1.20 [0.67, 2.14]
Test for heterogeneity: tau^2=0.02; chi^2=53.43, df=29, P=0.004; I^2=49%<br>Test for overall unadjusted random effect: Z=5.27, P<0.001					1.30 [1.18, 1.43]	-
Test for heterogeneity: tau^2=0.02; chi^2=49.61, df=29, P=0.010; I^2=44%<br>Test for overall adjusted random effect: Z=4.77, P<0.001					-	1.25 [1.14, 1.37]

Supplementary Figure 4: Meta-analysis comparing prevalence of wasting in children with and without disabilities

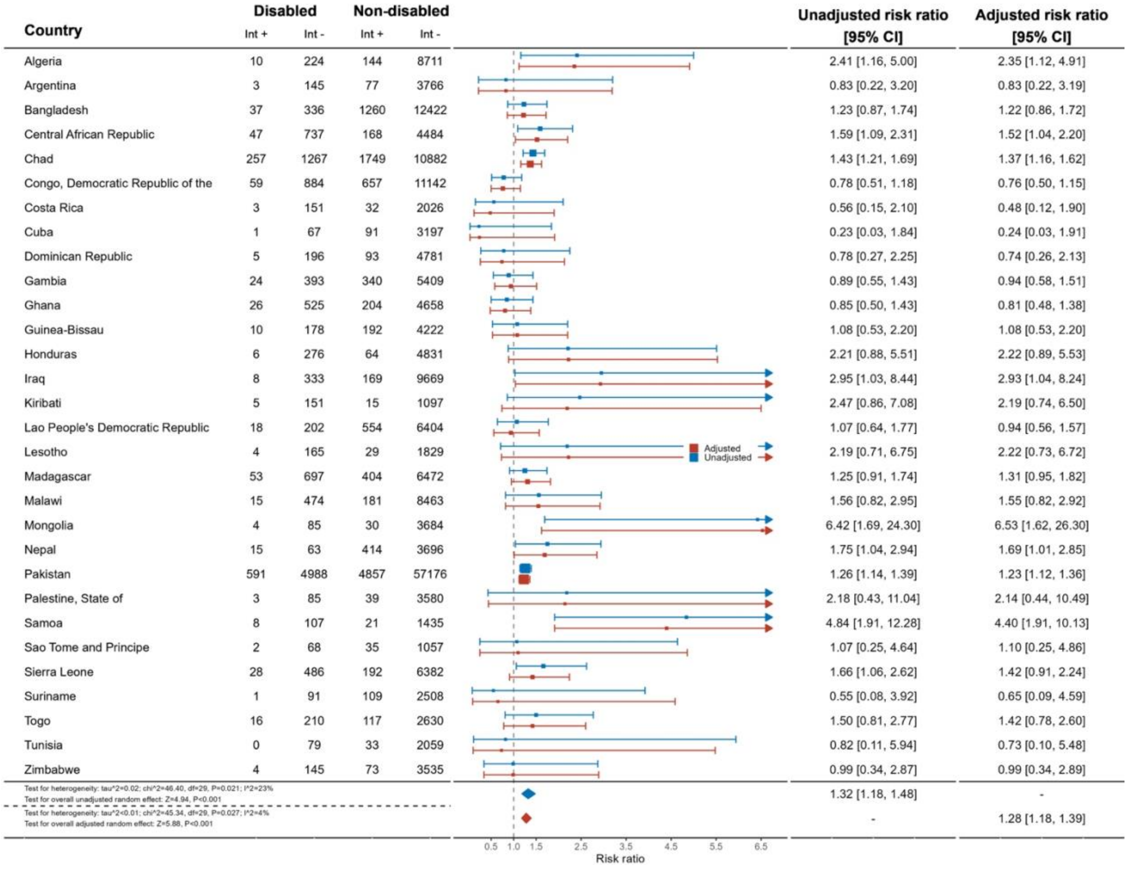

Supplementary Figure 5: Meta-analysis comparing prevalence of wasting in girls with and without disabilities

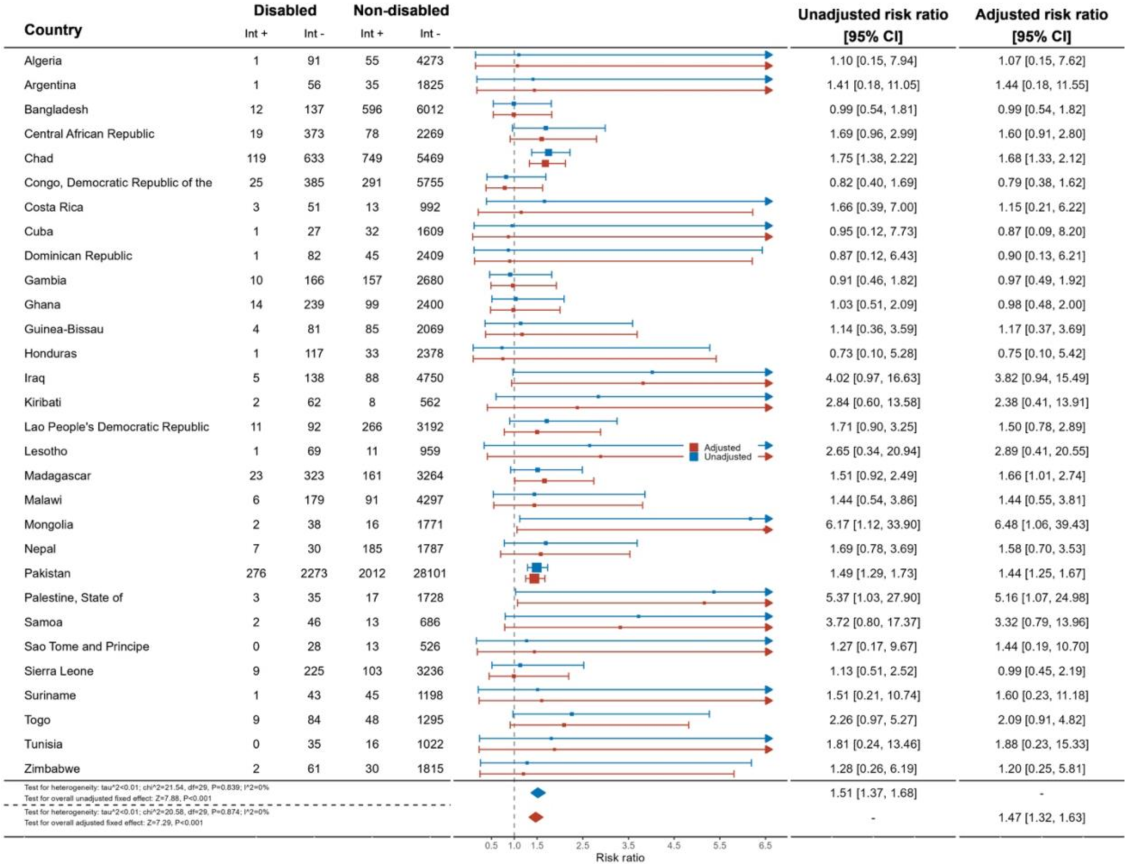

Country	Disabled Int +	Disabled Int -	Non-disabled Int +	Non-disabled Int -	Unadjusted risk ratio [95% CI]	Adjusted risk ratio [95% CI]
Algeria	1	91	55	4273	1.10 [0.15, 7.94]	1.07 [0.15, 7.62]
Argentina	1	56	35	1825	1.41 [0.18, 11.05]	1.44 [0.18, 11.55]
Bangladesh	12	137	596	6012	0.99 [0.54, 1.81]	0.99 [0.54, 1.82]
Central African Republic	19	373	78	2269	1.69 [0.96, 2.99]	1.60 [0.91, 2.80]
Chad	119	633	749	5469	1.75 [1.38, 2.22]	1.68 [1.33, 2.12]
Congo, Democratic Republic of the	25	385	291	5755	0.82 [0.40, 1.69]	0.79 [0.38, 1.62]
Costa Rica	3	51	13	992	1.66 [0.39, 7.00]	1.15 [0.21, 6.22]
Cuba	1	27	32	1609	0.95 [0.12, 7.73]	0.87 [0.09, 8.20]
Dominican Republic	1	82	45	2409	0.87 [0.12, 6.43]	0.90 [0.13, 6.21]
Gambia	10	166	157	2680	0.91 [0.46, 1.82]	0.97 [0.49, 1.92]
Ghana	14	239	99	2400	1.03 [0.51, 2.09]	0.98 [0.48, 2.00]
Guinea-Bissau	4	81	85	2069	1.14 [0.36, 3.59]	1.17 [0.37, 3.69]
Honduras	1	117	33	2378	0.73 [0.10, 5.28]	0.75 [0.10, 5.42]
Iraq	5	138	88	4750	4.02 [0.97, 16.63]	3.82 [0.94, 15.49]
Kiribati	2	62	8	562	2.84 [0.60, 13.58]	2.38 [0.41, 13.91]
Lao People's Democratic Republic	11	92	266	3192	1.71 [0.90, 3.25]	1.50 [0.78, 2.89]
Lesotho	1	69	11	959	2.65 [0.34, 20.94]	2.89 [0.41, 20.55]
Madagascar	23	323	161	3264	1.51 [0.92, 2.49]	1.66 [1.01, 2.74]
Malawi	6	179	91	4297	1.44 [0.54, 3.86]	1.44 [0.55, 3.81]
Mongolia	2	38	16	1771	6.17 [1.12, 33.90]	6.48 [1.06, 39.43]
Nepal	7	30	185	1787	1.69 [0.78, 3.69]	1.58 [0.70, 3.53]
Pakistan	276	2273	2012	28101	1.49 [1.29, 1.73]	1.44 [1.25, 1.67]
Palestine, State of	3	35	17	1728	5.37 [1.03, 27.90]	5.16 [1.07, 24.98]
Samoa	2	46	13	686	3.72 [0.80, 17.37]	3.32 [0.79, 13.96]
Sao Tome and Principe	0	28	13	526	1.27 [0.17, 9.67]	1.44 [0.19, 10.70]
Sierra Leone	9	225	103	3236	1.13 [0.51, 2.52]	0.99 [0.45, 2.19]
Suriname	1	43	45	1198	1.51 [0.21, 10.74]	1.60 [0.23, 11.18]
Togo	9	84	48	1295	2.26 [0.97, 5.27]	2.09 [0.91, 4.82]
Tunisia	0	35	16	1022	1.81 [0.24, 13.46]	1.88 [0.23, 15.33]
Zimbabwe	2	61	30	1815	1.28 [0.26, 6.19]	1.20 [0.25, 5.81]
Test for heterogeneity: tau^2<0.01; chi^2=21.54, df=29, P=0.839; I^2=0%<br>Test for overall unadjusted fixed effect: Z=7.88, P<0.001					1.51 [1.37, 1.68]	-
Test for heterogeneity: tau^2<0.01; chi^2=20.58, df=29, P=0.874; I^2=0%<br>Test for overall adjusted fixed effect: Z=7.29, P<0.001					-	1.47 [1.32, 1.63]

Supplementary Figure 6: Meta-analysis comparing prevalence of wasting in boys with and without disabilities

Country	Disabled Int +	Disabled Int -	Non-disabled Int +	Non-disabled Int -	Unadjusted risk ratio [95% CI]	Adjusted risk ratio [95% CI]
Algeria	9	133	89	4438	2.80 [1.25, 6.25]	2.73 [1.21, 6.17]
Argentina	2	89	42	1941	0.55 [0.12, 2.47]	0.52 [0.11, 2.45]
Bangladesh	25	199	664	6410	1.37 [0.90, 2.09]	1.34 [0.88, 2.04]
Central African Republic	28	364	90	2215	1.49 [0.92, 2.43]	1.47 [0.90, 2.40]
Chad	138	634	1000	5413	1.19 [0.94, 1.51]	1.15 [0.90, 1.46]
Congo, Democratic Republic of the	34	499	366	5387	0.72 [0.43, 1.21]	0.72 [0.43, 1.19]
Costa Rica	0	100	19	1034	0.19 [0.02, 1.46]	0.17 [0.02, 1.29]
Cuba	0	40	59	1588	0.49 [0.06, 4.27]	0.55 [0.06, 4.74]
Dominican Republic	4	114	48	2372	0.70 [0.21, 2.36]	0.63 [0.18, 2.17]
Gambia	14	227	183	2729	0.86 [0.45, 1.65]	0.91 [0.47, 1.76]
Ghana	12	286	105	2258	0.72 [0.34, 1.50]	0.69 [0.33, 1.46]
Guinea-Bissau	6	97	107	2153	1.02 [0.42, 2.50]	1.02 [0.42, 2.49]
Honduras	5	159	31	2453	3.62 [1.28, 10.23]	3.56 [1.26, 10.11]
Iraq	3	195	81	4919	2.01 [0.52, 7.80]	2.15 [0.56, 8.26]
Kiribati	3	89	7	535	2.24 [0.54, 9.35]	2.18 [0.54, 8.77]
Lao People's Democratic Republic	7	110	288	3212	0.62 [0.28, 1.35]	0.55 [0.25, 1.22]
Lesotho	3	96	18	870	1.80 [0.48, 6.77]	1.74 [0.47, 6.49]
Madagascar	30	374	243	3208	1.08 [0.70, 1.66]	1.12 [0.73, 1.72]
Malawi	9	295	90	4166	1.58 [0.69, 3.61]	1.57 [0.69, 3.61]
Mongolia	2	47	14	1913	6.43 [1.35, 30.54]	12.33 [2.69, 56.59]
Nepal	8	33	229	1909	1.81 [0.91, 3.58]	1.79 [0.92, 3.49]
Pakistan	315	2715	2845	29075	1.10 [0.96, 1.26]	1.09 [0.95, 1.25]
Palestine, State of	0	50	22	1852	2.36 [0.32, 17.40]	2.48 [0.31, 19.86]
Samoa	6	61	8	749	7.44 [2.44, 22.75]	6.97 [2.63, 18.46]
Sao Tome and Principe	2	40	22	531	1.26 [0.29, 5.52]	1.26 [0.28, 5.74]
Sierra Leone	19	261	89	3146	2.39 [1.38, 4.14]	1.95 [1.12, 3.41]
Suriname	0	48	64	1310	0.27 [0.04, 2.04]	0.31 [0.04, 2.34]
Togo	7	126	69	1335	1.01 [0.41, 2.48]	0.98 [0.40, 2.36]
Tunisia	0	44	17	1037	1.51 [0.20, 11.26]	1.09 [0.15, 8.04]
Zimbabwe	2	84	43	1720	0.79 [0.18, 3.35]	0.86 [0.20, 3.68]
Test for heterogeneity: tau^2=0.10; chi^2=53.98, df=29, P=0.003; I^2=54%<br>Test for overall unadjusted random effect: Z=2.49, P=0.013					1.28 [1.05, 1.56]	-
Test for heterogeneity: tau^2=0.14; chi^2=58.34, df=29, P=0.001; I^2=61%<br>Test for overall adjusted random effect: Z=2.31, P=0.021					-	1.28 [1.04, 1.58]

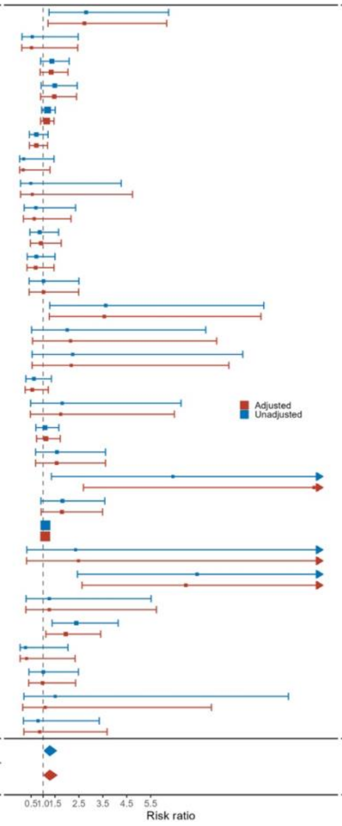

Supplementary Figure 7: Meta-analysis comparing prevalence of stunting in children with and without disabilities

Country	Disabled Int +	Disabled Int -	Non-disabled Int +	Non-disabled Int -	Unadjusted risk ratio [95% CI]	Adjusted risk ratio [95% CI]
Algeria	36	198	849	8006	1.38 [0.90, 2.10]	1.35 [0.89, 2.06]
Argentina	29	119	348	3495	1.17 [0.69, 1.96]	1.13 [0.67, 1.90]
Bangladesh	139	234	3939	9743	1.28 [1.10, 1.50]	1.22 [1.06, 1.42]
Central African Republic	379	405	2021	2631	1.12 [1.03, 1.23]	1.09 [1.00, 1.19]
Chad	717	807	5154	7477	1.14 [1.06, 1.23]	1.10 [1.02, 1.18]
Congo, Democratic Republic of the	518	425	5864	5935	1.18 [1.06, 1.31]	1.15 [1.04, 1.27]
Costa Rica	13	141	128	1930	1.13 [0.47, 2.73]	1.19 [0.51, 2.78]
Cuba	8	60	226	3062	5.47 [1.86, 16.03]	5.58 [1.98, 15.73]
Dominican Republic	26	175	257	4617	2.39 [1.37, 4.16]	1.99 [1.13, 3.50]
Gambia	133	284	1292	4457	1.48 [1.23, 1.77]	1.27 [1.07, 1.52]
Ghana	105	446	936	3926	0.95 [0.74, 1.23]	0.88 [0.68, 1.14]
Guinea-Bissau	70	118	1233	3181	1.29 [1.04, 1.61]	1.26 [1.02, 1.57]
Honduras	89	193	1095	3800	1.45 [1.17, 1.79]	1.39 [1.14, 1.70]
Iraq	60	281	995	8843	1.72 [1.23, 2.40]	1.56 [1.10, 2.20]
Kiribati	42	114	196	916	1.57 [1.16, 2.13]	1.49 [1.10, 2.02]
Lao People's Democratic Republic	126	94	2761	4197	1.50 [1.29, 1.73]	1.24 [1.07, 1.44]
Lesotho	68	101	642	1216	1.17 [0.93, 1.49]	1.09 [0.86, 1.38]
Madagascar	353	397	2944	3932	1.05 [0.96, 1.16]	1.02 [0.93, 1.13]
Malawi	202	287	3011	5633	1.11 [0.97, 1.28]	1.07 [0.94, 1.23]
Mongolia	20	69	412	3302	1.18 [0.64, 2.17]	1.12 [0.60, 2.09]
Nepal	33	45	1468	2642	1.31 [0.97, 1.76]	1.27 [0.96, 1.67]
Pakistan	2804	2775	25870	36163	1.17 [1.13, 1.21]	1.15 [1.11, 1.19]
Palestine, State of	7	81	263	3356	0.99 [0.42, 2.35]	0.87 [0.37, 2.07]
Samoa	14	101	101	1355	1.97 [1.12, 3.46]	1.69 [0.95, 3.01]
Sao Tome and Principe	8	62	146	946	0.89 [0.44, 1.82]	0.81 [0.40, 1.66]
Sierra Leone	207	307	2044	4530	1.29 [1.13, 1.48]	1.22 [1.07, 1.40]
Suriname	5	87	168	2449	1.57 [0.61, 4.03]	1.34 [0.55, 3.24]
Togo	65	161	759	1988	1.05 [0.80, 1.38]	1.08 [0.82, 1.41]
Tunisia	10	69	166	1926	1.69 [0.91, 3.13]	1.55 [0.82, 2.92]
Zimbabwe	41	108	874	2734	1.17 [0.87, 1.56]	1.06 [0.79, 1.43]
Test for heterogeneity: tau^2=0.01; chi^2=63.44, df=29, P<0.001; I^2=62%<br>Test for overall unadjusted random effect: Z=7.49, P<0.001					1.24 [1.18, 1.32]	-
Test for heterogeneity: tau^2<0.01; chi^2=45.82, df=29, P=0.024; I^2=28%<br>Test for overall adjusted random effect: Z=7.43, P<0.001					-	1.16 [1.11, 1.20]

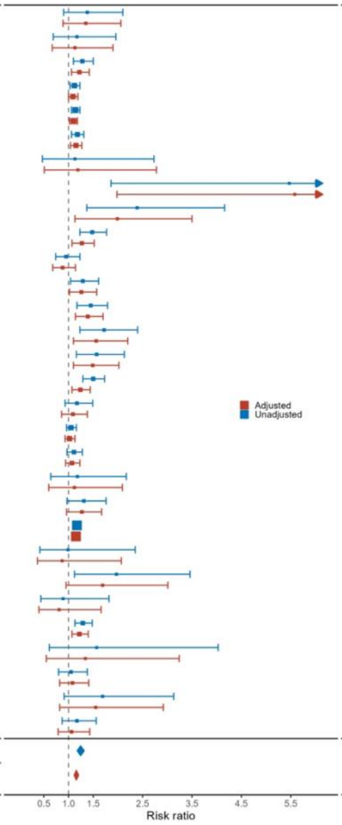

Supplementary Figure 8: Meta-analysis comparing prevalence of stunting in girls with and without disabilities

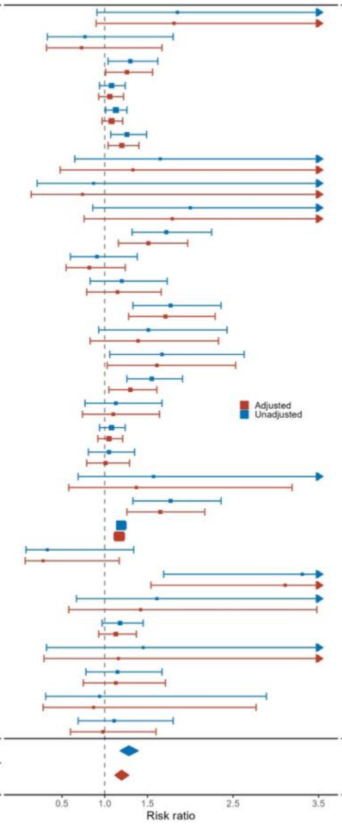

Country	Disabled Int +	Disabled Int -	Non-disabled Int +	Non-disabled Int -	Unadjusted risk ratio [95% CI]	Adjusted risk ratio [95% CI]
Algeria	13	79	371	3957	1.85 [0.91, 3.77]	1.81 [0.90, 3.66]
Argentina	11	46	184	1676	0.77 [0.33, 1.80]	0.73 [0.32, 1.67]
Bangladesh	58	91	1983	4625	1.30 [1.04, 1.62]	1.26 [1.01, 1.56]
Central African Republic	169	223	977	1370	1.08 [0.94, 1.24]	1.06 [0.93, 1.22]
Chad	358	394	2502	3716	1.13 [1.01, 1.26]	1.08 [0.97, 1.21]
Congo, Democratic Republic of the	206	204	2880	3166	1.26 [1.07, 1.49]	1.20 [1.04, 1.40]
Costa Rica	7	47	66	939	1.65 [0.65, 4.14]	1.33 [0.48, 3.66]
Cuba	3	25	110	1531	0.87 [0.21, 3.56]	0.74 [0.14, 3.81]
Dominican Republic	10	73	135	2319	2.00 [0.86, 4.65]	1.79 [0.76, 4.21]
Gambia	60	116	614	2223	1.72 [1.32, 2.25]	1.51 [1.16, 1.97]
Ghana	41	212	429	2070	0.91 [0.60, 1.38]	0.82 [0.55, 1.24]
Guinea-Bissau	26	59	567	1587	1.20 [0.83, 1.73]	1.15 [0.79, 1.66]
Honduras	39	79	528	1883	1.77 [1.33, 2.36]	1.71 [1.28, 2.29]
Iraq	31	112	626	4212	1.51 [0.93, 2.43]	1.39 [0.83, 2.33]
Kiribati	19	45	100	470	1.67 [1.06, 2.63]	1.61 [1.03, 2.53]
Lao People's Democratic Republic	60	43	1357	2101	1.55 [1.26, 1.91]	1.30 [1.05, 1.61]
Lesotho	26	44	319	651	1.13 [0.77, 1.67]	1.10 [0.74, 1.64]
Madagascar	158	188	1411	2014	1.08 [0.94, 1.24]	1.05 [0.92, 1.21]
Malawi	68	117	1424	2964	1.05 [0.81, 1.35]	1.01 [0.79, 1.29]
Mongolia	11	29	168	1619	1.57 [0.69, 3.61]	1.37 [0.58, 3.19]
Nepal	20	17	721	1251	1.77 [1.33, 2.36]	1.65 [1.26, 2.17]
Pakistan	1317	1232	12665	17448	1.19 [1.14, 1.25]	1.17 [1.11, 1.23]
Palestine, State of	2	36	140	1605	0.33 [0.08, 1.34]	0.28 [0.07, 1.17]
Samoa	10	38	47	652	3.31 [1.69, 6.50]	3.11 [1.54, 6.26]
Sao Tome and Principe	5	23	61	478	1.61 [0.67, 3.87]	1.42 [0.58, 3.48]
Sierra Leone	97	137	1097	2242	1.18 [0.97, 1.45]	1.13 [0.93, 1.37]
Suriname	2	42	64	1179	1.45 [0.32, 6.51]	1.16 [0.29, 4.72]
Togo	30	63	349	994	1.15 [0.78, 1.67]	1.13 [0.75, 1.71]
Tunisia	3	32	88	950	0.94 [0.31, 2.89]	0.87 [0.28, 2.77]
Zimbabwe	15	48	406	1439	1.11 [0.69, 1.80]	0.98 [0.60, 1.60]
Test for heterogeneity: tau^2=0.02; chi^2=57.09, df=29, P=0.001; I^2=61%<br>Test for overall unadjusted random effect: Z=5.89, P<0.001					1.28 [1.18, 1.39]	-
Test for heterogeneity: tau^2=0.01; chi^2=47.40, df=29, P=0.017; I^2=44%<br>Test for overall adjusted random effect: Z=5.18, P<0.001					-	1.20 [1.12, 1.28]

Supplementary Figure 9: Meta-analysis comparing prevalence of stunting in boys with and without disabilities

Country	Disabled Int +	Disabled Int -	Non-disabled Int +	Non-disabled Int -	Unadjusted risk ratio [95% CI]	Adjusted risk ratio [95% CI]
Algeria	23	119	478	4049	1.10 [0.67, 1.81]	1.08 [0.65, 1.79]
Argentina	18	73	164	1819	1.78 [0.96, 3.31]	1.76 [0.93, 3.33]
Bangladesh	81	143	1956	5118	1.29 [1.06, 1.59]	1.21 [1.00, 1.47]
Central African Republic	210	182	1044	1261	1.16 [1.04, 1.30]	1.12 [1.00, 1.26]
Chad	359	413	2652	3761	1.16 [1.05, 1.28]	1.11 [1.01, 1.22]
Congo, Democratic Republic of the	312	221	2984	2769	1.11 [0.96, 1.27]	1.09 [0.96, 1.25]
Costa Rica	6	94	62	991	0.89 [0.23, 3.43]	1.10 [0.33, 3.70]
Cuba	5	35	116	1531	8.23 [3.28, 20.69]	7.55 [3.52, 16.17]
Dominican Republic	16	102	122	2298	2.67 [1.29, 5.52]	2.06 [0.98, 4.36]
Gambia	73	168	678	2234	1.30 [1.01, 1.67]	1.11 [0.88, 1.41]
Ghana	64	234	507	1856	0.97 [0.70, 1.34]	0.91 [0.66, 1.27]
Guinea-Bissau	44	59	666	1594	1.36 [1.04, 1.77]	1.35 [1.05, 1.73]
Honduras	50	114	567	1917	1.23 [0.91, 1.66]	1.17 [0.89, 1.54]
Iraq	29	169	369	4631	2.16 [1.35, 3.46]	1.91 [1.20, 3.04]
Kiribati	23	69	96	446	1.50 [1.00, 2.26]	1.44 [0.96, 2.17]
Lao People's Democratic Republic	66	51	1404	2096	1.45 [1.18, 1.78]	1.20 [0.98, 1.46]
Lesotho	42	57	323	565	1.20 [0.88, 1.62]	1.07 [0.80, 1.43]
Madagascar	195	209	1533	1918	1.03 [0.90, 1.18]	1.01 [0.89, 1.15]
Malawi	134	170	1587	2669	1.12 [0.95, 1.31]	1.08 [0.93, 1.27]
Mongolia	9	40	244	1683	0.90 [0.36, 2.24]	0.90 [0.36, 2.24]
Nepal	13	28	747	1391	0.84 [0.49, 1.43]	0.85 [0.51, 1.41]
Pakistan	1487	1543	13205	18715	1.15 [1.09, 1.20]	1.14 [1.09, 1.19]
Palestine, State of	5	45	123	1751	1.74 [0.65, 4.64]	1.57 [0.59, 4.17]
Samoa	4	63	54	703	1.00 [0.36, 2.82]	0.84 [0.31, 2.32]
Sao Tome and Principe	3	39	85	468	0.54 [0.17, 1.73]	0.49 [0.16, 1.54]
Sierra Leone	110	170	947	2288	1.41 [1.17, 1.69]	1.34 [1.12, 1.61]
Suriname	3	45	104	1270	1.69 [0.52, 5.54]	1.57 [0.52, 4.74]
Togo	35	98	410	994	0.98 [0.67, 1.42]	1.03 [0.72, 1.47]
Tunisia	7	37	78	976	2.54 [1.22, 5.28]	2.13 [1.00, 4.54]
Zimbabwe	26	60	468	1295	1.16 [0.80, 1.67]	1.06 [0.73, 1.55]
Test for heterogeneity: tau^2=0.01; chi^2=59.81, df=29, P=0.001; I^2=35%<br>Test for overall unadjusted random effect: Z=6.57, P<0.001					1.21 [1.14, 1.28]	-
Test for heterogeneity: tau^2<0.01; chi^2=53.49, df=29, P=0.004; I^2=0%<br>Test for overall adjusted random effect: Z=7.95, P<0.001					-	1.14 [1.10, 1.17]

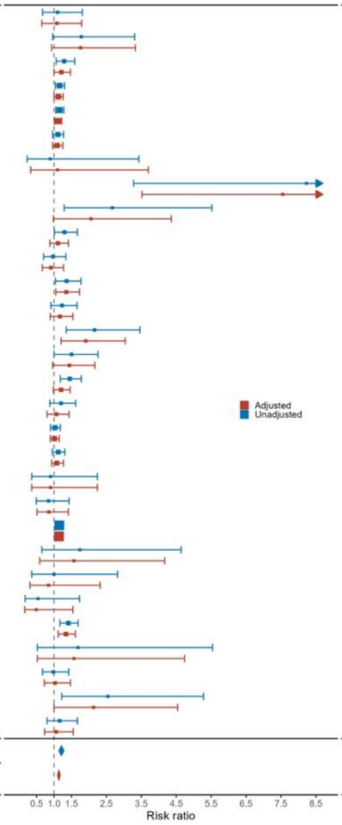

Supplement: Supplementary data [file bmjnph-2023-000779supp001.pdf]
